# Pulse oximetry-derived respiratory rate in general care floor patients

**DOI:** 10.1007/s10877-014-9575-5

**Published:** 2014-05-06

**Authors:** Paul S. Addison, James N. Watson, Michael L. Mestek, James P. Ochs, Alberto A. Uribe, Sergio D. Bergese

**Affiliations:** 1 Covidien Respiratory and Monitoring Solutions, Edinburgh, Scotland, UK; 2 Covidien Respiratory and Monitoring Solutions, Boulder, CO USA; 3 Department of Anesthesiology, The Ohio State University Wexner Medical Center, Columbus, OH USA; 4 Departments of Anesthesiology and Neurological Surgery, The Ohio State University Medical Center, Columbus, OH USA

**Keywords:** Respiratory rate, Pulse oximeter, Continuous monitoring, Low acuity monitoring

## Abstract

Respiratory rate is recognized as a clinically important parameter for monitoring respiratory status on the general care floor (GCF). Currently, intermittent manual assessment of respiratory rate is the standard of care on the GCF. This technique has several clinically-relevant shortcomings, including the following: (1) it is not a continuous measurement, (2) it is prone to observer error, and (3) it is inefficient for the clinical staff. We report here on an algorithm designed to meet clinical needs by providing respiratory rate through a standard pulse oximeter. Finger photoplethysmograms were collected from a cohort of 63 GCF patients monitored during free breathing over a 25-min period. These were processed using a novel in-house algorithm based on continuous wavelet-transform technology within an infrastructure incorporating confidence-based averaging and logical decision-making processes. The computed oximeter respiratory rates (RR_oxi_) were compared to an end-tidal CO_2_ reference rate (RR_ETCO2_). RR_ETCO2_ ranged from a lowest recorded value of 4.7 breaths per minute (brpm) to a highest value of 32.0 brpm. The mean respiratory rate was 16.3 brpm with standard deviation of 4.7 brpm. Excellent agreement was found between RR_oxi_ and RR_ETCO2_, with a mean difference of −0.48 brpm and standard deviation of 1.77 brpm. These data demonstrate that our novel respiratory rate algorithm is a potentially viable method of monitoring respiratory rate in GCF patients. This technology provides the means to facilitate continuous monitoring of respiratory rate, coupled with arterial oxygen saturation and pulse rate, using a single non-invasive sensor in low acuity settings.

## Introduction

Respiratory rate (RR) is well known to be a clinically important parameter owing to the fact that it provides important information pertaining to many aspects of a patient’s respiratory status. Frequently, a change in RR is one of the earliest and more important indicators that precedes major clinical manifestations of serious complications such as respiratory tract infections, respiratory depression associated with opioid consumption, anaesthesia and/or sedation, as well as respiratory failure [5]. Accordingly, the monitoring of RR is of paramount importance in several clinical conditions, particularly in settings where direct and close clinician supervision is minimal, such as the general care floor (GCF).

In current clinical practice, the standard of care technique for monitoring RR is intermittent, manual observation. This technique consists of visual assessment of patient breathing for a one-minute period of time to establish RR via a manual count. While manual observation is currently in widespread use in many patient care settings, there are several clinically important shortcomings associated with this approach. For example, manual counting requires clinical staff intervention, which often leads to low rates of compliance for RR monitoring. Furthermore, it is prone to significant errors that may stem from a number of sources, including failure to observe distinct breaths; counting and rounding errors; and, not least, erroneous respiration due to self-conscious breathing caused by patient-clinician interaction [14].

Several technological approaches have been advanced in the attempt to address the limitations associated with manual observation of RR. Examples of these technologies include several different continuous monitoring devices such as end-tidal CO_2_ (ETCO_2_), ECG-based trans-thoracic impedance systems, nasal thermistors, and abdominal and chest bands (i.e. respiratory inductance plethysmography) [6]. However, continuous measurements using these techniques all involve costly, specialized, and intrusive equipment that ultimately is limiting to their practical application in many care settings, particularly in low acuity settings such as the GCF. In addition to the technical challenges and expense of the current continuous measurement options, such devices may be cumbersome for the patient and thus patient compliance may be low due to physical discomfort. Indeed, it has previously been demonstrated that when ETCO_2_ monitoring is employed on the GCF, many patients remove the nasal cannula due to physical annoyance [7]. Evaluation of the current methodologies, both manual observation and technological approaches, reveals that there is an obvious need for an alternative methodology for monitoring RR that overcomes the deficiencies of these approaches. An ideal clinical solution would be one that is continuous in nature, non-invasive, simple to operate, unobtrusive, clinically acceptable, and robust in the presence of signal interference.

One such non-invasive continuous monitoring technology candidate that is affordable, user-friendly, and already accepted in clinical practice is pulse oximetry. Recent evidence indicates that the measurement of RR from the pulse oximeter signal, or photoplethysmogram (PPG), may be possible. Numerous groups have previously demonstrated that evaluating the respiratory-related fluctuations from the PPG signal is both a biologically plausible and technically attainable approach to obtaining RR using a variety of methods including: inspection of the respiratory oscillations in the filtered PPG [15, 25, 27, 31–33]; frequency spectra-based approaches [28, 35]; frequency-based smart fusion approaches using multiple modulations [18]; independent component analysis [36]; short-time Fourier transform analysis [34]; neural networks [26]; variable frequency complex demodulation methods [13, 21]; autoregressive models [23, 24, 29]; pulse width variability [17]; and approaches based on the continuous wavelet transform by our own group [2, 10–12, 22, 30].

This cumulative body of evidence strongly suggests the possibility of deriving RR from a single combined sensing system that leverages standard pulse oximetry. A technological approach providing these metrics from a single sensor would yield tremendous clinical utility in a manner that is cost effective and efficient from a workflow perspective. We have shown in a recent study [2] that the respiratory rate determined from our in house algorithm (RR_oxi_) represents a potentially viable technology for the measurement of RR in healthy subjects. The algorithm has the necessary filtering, logic and decision making processes required to provide a fully-automated technology capable of coping with the extremes of data characteristics in the clinical environment and ultimately provide a clinically useful number for display. The purpose of the follow-up study we report here was to demonstrate the viability of RR_oxi_ in a GCF patient population.

## Methods and materials

### The respiratory rate algorithm (RR_oxi_)

**Fig. 1:**
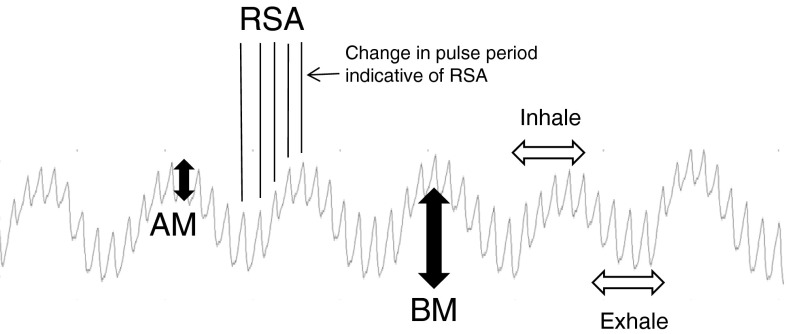
A segment of PPG exhibiting the three modulations. *BM* baseline modulation (cardiac pulses riding on *top* of baseline modulation), *AM* amplitude modulation (cardiac pulse amplitudes varying over respiratory cycle), *RSA* respiratory sinus arrhythmia (pulse period varying over respiratory cycle). Regions of inhalation and exhalation are shown schematically on one respiratory cycle

Respiratory activity may cause the PPG to contain three fundamental waveform modulations [2, 18, 47]. These can be seen in the example signal segment in Fig. 1 and described as follows:

1. Baseline (DC) Modulation: caused by changes in venous return secondary to changes in intrathoracic pressure throughout the respiratory cycle. During inspiration, the decrease in intrathoracic pressure results in a small decrease in central venous pressure increasing venous return. The opposite occurs during expiration. As more blood is shunted from the low pressure venous system at the probe site and the venous bed cyclically fills and drains, the baseline is modulated accordingly (‘BM’ in Fig. 1).
2. Pulse Amplitude Modulation: Respiratory-related changes in intrathoracic pressure alter cardiac function. Principally, this stems from decreased left ventricular stroke volume during inspiration, leading to decreased pulse amplitude during this phase of respiration (‘AM’ in Fig. 1).
3. Respiratory Sinus Arrhythmia (RSA): This is a variation in heart rate that occurs throughout the respiratory cycle. Specifically, it has been well-documented that heart rate increases during inspiration and decreases during expiration. However, the presence of RSA is influenced by several factors including age, disease status, and physical fitness. While the precise mechanisms of RSA remain controversial, in general, it is a result of autonomic nervous system activity fluctuation during respiration (‘RSA’ in Fig. 1).

We have developed a powerful signal processing methodology that can extract respiratory information from the PPG. This is embodied within our RR algorithm (RR_oxi_) that optimizes the extraction of respiratory information from within the PPG signal. This is achieved by deriving a series of new characterizing signals which are optimally configured to enhance respiratory information content. These are fed into the main analysis engine which processes the characterizing signals in order to determine a RR. The analysis engine incorporates advanced signal processing techniques based on continuous wavelet transform methods [1]. The wavelet transform of a signal *x*(*t*) is defined as:

1 $$T(a,b) = \frac{1}{\sqrt a }\int\limits_{ - \infty }^{ + \infty } {x(t)\psi^{*} \left( {\frac{t - b}{a}} \right)} dt$$

where ψ*(t) is the complex conjugate of the wavelet function ψ*(t)*, *a* is the dilation or scale parameter of the wavelet, *b* is the location parameter of the wavelet, *t* is time and *x(t)* is the signal under investigation: this may be the PPG or secondary signals derived from the PPG. In our work we employ tunable complete Morlet wavelets (1) of the form:

2 $$\psi (t) = \frac{1}{{\sqrt[4]{\pi }}}\left( {\mathop e\nolimits^{{i\omega_{o} t}} - \mathop e\nolimits^{{ - \frac{{\omega_{o}^{2} }}{2}}} } \right)\mathop e\nolimits^{{ - \frac{{t^{2} }}{2}}}$$

where ω_o_ is the central frequency of the mother wavelet. The second term in the brackets is known as the correction term, as it corrects for the non-zero mean of the complex sinusoid of the first term. The RR_oxi_ algorithm iterates every 5 s, deriving an RR from the previous 45-s segment of infrared PPG. These current rates are averaged further with the previously displayed rate, and continue through additional logic before displaying a final reported rate to the user. We provide additional detailed information on the algorithm in references [2, 48–53] including the state machine-driven logic used to determine whether to report the information to the end user or blank out the display [2].

### Study details

#### Subjects

Subsequent to IRB approval, the study was conducted at The Ohio State University Medical Center in Columbus, OH, USA. A cohort of 63 adult patients was recruited for the trial (33 male and 30 female). RR was determined from the data acquired from all subjects.

The study exclusion criteria were:

- Contact allergies that may cause a reaction to standard adhesive materials found in the sensors used.
- Abnormalities that may prevent proper application of the pulse oximeter probe.
- Previous injury or co-morbidity to fingers or hands that may change blood flow and vascular supply.
- Pregnant or lactating women.

The latter being a standard criterion for the site where most of the studies exclude pregnant or lactating woman.

#### Protocol

The data were acquired using a standard Nell-1 oximeter OEM module with a Nellcor Max-A disposable probe attached firmly to the index finger of the right hand. A Datex-Ohmeda CardioCap/S5 device was used to record an end-tidal CO_2_ signal from the patient using a nasal cannula. Once the subject was comfortable with the equipment, the PPG signal was acquired for a duration of approximately 25 min. A spontaneous breathing protocol was conducted whereby the subjects were asked to relax and breathe naturally. In addition, research clinical personnel recorded any external artifacts or subjects’ movements during data collection to ensure data quality. The patients were observed but no other instructions were given.

## Results

Participant characteristics are detailed in Table 1. The participants exhibited a wide range of medical conditions. These are detailed in Table 2.

**Table 1:** Selected subject characteristics

Variable	Mean ± SD	Min	Max
Age (year)	55 ± 17	24	89
Weight (kg)	92 ± 26	45	170
Height (cm)	170 ± 10	150	198
BMI (kg/m^2^)	31 ± 9	15	61

**Table 2:** Subject medical condition classification

Medical condition	Number	Percentage (%)
*Respiratory*		
Asthma	4	6.3
Chronic obstructive pulmonary disease	8	12.7
Dyspnea	2	3.2
Obstructive sleep apnea	7	11.1
Pneumonia	2	3.2
*Cardiovascular*		
Aortic stenosis	3	4.8
Coronary artery disease	8	12.7
Heart failure	5	7.9
Hypertension	24	38.1
Stroke	3	4.8
*Metabolic/autonomic*		
Hyperlipidemia	9	14.3
Obesity	31	49.2
Neuropathy	12	19.0
Type II diabetes mellitus	15	23.8
*Renal*		
End stage renal disease	2	3.2

**Fig. 2:**
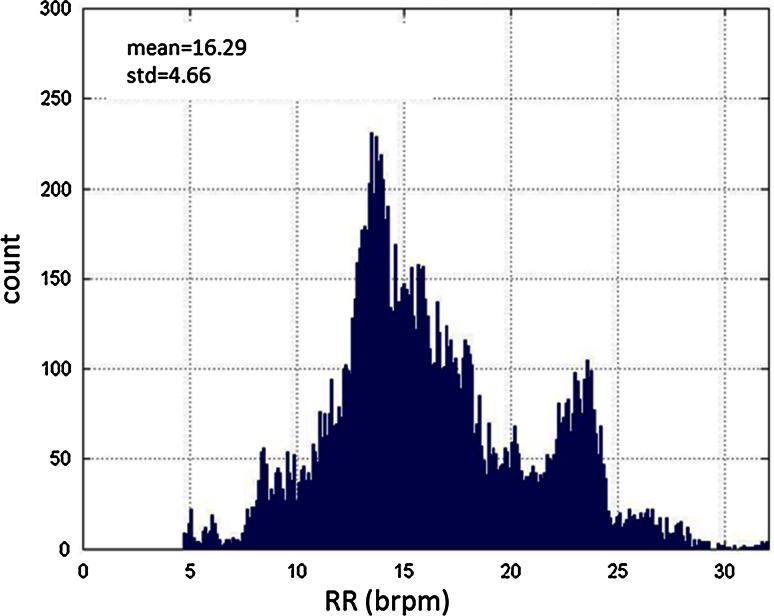
Distribution of breathing rates (RR_ETCO2_) of GCF patients during the trial

Figure 2 contains the spread of the RR during the study. The histogram plot is comprised of 16,980 RR_ETCO2_ data points taken at 5-s increments over the whole population; that is, at each 5 s increment, the previous 45 s of data is used to determine RR_oxi_. RR_ETCO2_ ranged from a lowest recorded value of 4.7 brpm to a highest value of 32.0 brpm. The mean rate was 16.3 brpm with a standard deviation of 4.7 brpm. To ensure that we had the highest possible confidence in the ETCO_2_ reference, we took steps to eliminate regions of poor quality ETCO_2_ waveforms that might typically be included in normal device operation. For example, ETCO_2_ devices will often report through a degree of talking and motion induced artifact; however, the actual RR is often ambiguous in these regions. Therefore, in an effort to ensure a high confidence in the ETCO_2_ RR, we ignored these regions in the performance analysis. Of the regions tested, a rate was not computed from the RR_ETCO2_ for 17 % of the data due to poor or ambiguous signal quality or the device recalibrating.

**Fig. 3:**
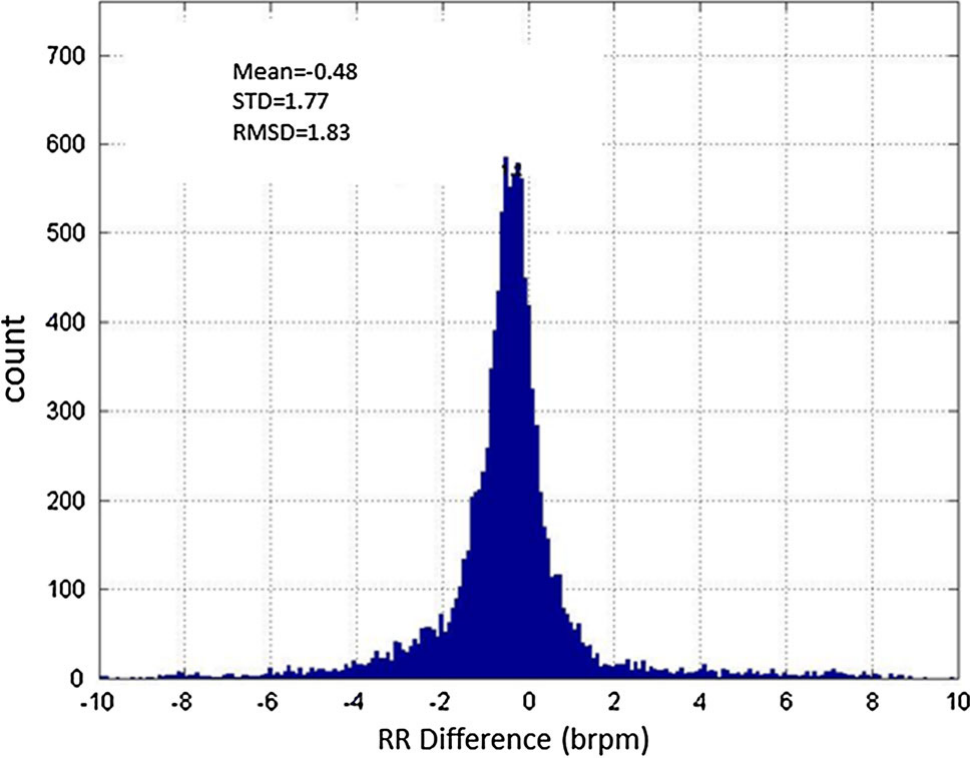
Distribution of differences between RR_ETCO2_ and RR_oxi_

**Fig. 4:**
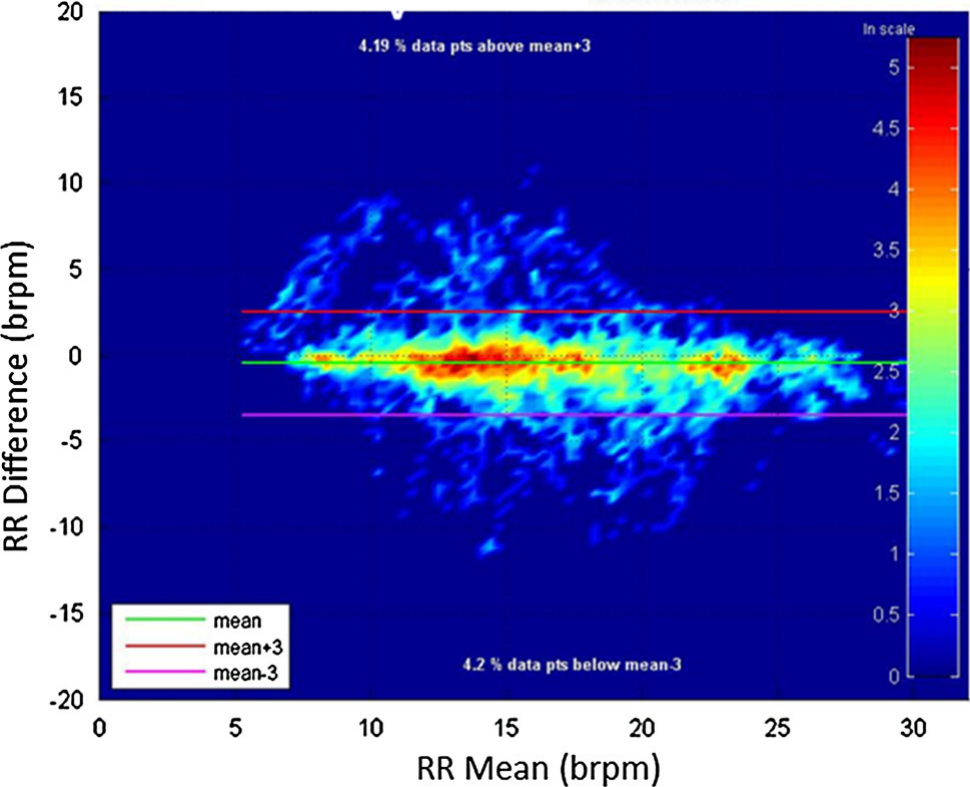
Bland-Altman density plot of the data (lowest density of points to highest density = *Dark Blue*, *Light Blue*, *Green*, *Yellow*, *Red*)

Figure 3 contains a histogram of the differences between RR_oxi_ and RR_ETCO2_. RR_ETCO2_ reported a rate 83.0 % of the time. RR_oxi_ reported a rate 92.4 % of the time. The overlap of non-reporting times for the two RRs was 1.2 %. The mean difference between the rates was −0.48 brpm with a standard deviation of 1.77 brpm. The root mean square deviation (RMSD) was 1.83 brpm (Pulse oximetry-based parameters SpO_2_ and pulse rate use RMSD as a measure that combines both bias (mean error) and precision (SD error) to give a measure of total error as per the ISO80601-2-61 standard [54]. We have adopted this in lieu of a standard for pulse oximetry-derived RR.) Figure 4 expands the view of the data further. The figure contains a Bland–Altman plot of the data. We have used a density scale of the data points to indicate the density of points contributing to the Bland–Altman plot. The mean and ±3 standard deviations of the data are plotted on the figure. We advocate these “density Bland–Altman” plots over the traditional method of simply plotting the data points for large sets. An appreciation of the number of points in a region is often difficult using the traditional method where large numbers of points may be plotted over each other, whereas the density plot provides a much clearer picture of where the majority of the data lies.

## Discussion

We have demonstrated an algorithm for the computation of RR from a standard, commercially available pulse oximetry system during spontaneous breathing in GCF patients in the GCF setting. RR_oxi_ was derived for data collected from a 63-subject cohort and compared to a reference rate, RR_ETCO2_. We found excellent agreement between the two with an RMSD of 1.83 brpm. Significantly, the algorithm was developed and tested using a wide range of in-hospital patient data [2, 55–62] and healthy subject data and therefore is not tuned specifically for GCF patients. This was done to mitigate overtraining on GCF data and to ensure that the algorithm has the ability to cope with as wide a range of situations in the field as possible. These results are in accordance with a previous study by our group of healthy volunteers [2]. Compared to this earlier study, there is a slight decrease in performance (mean 0.48 vs. 0.23 brpm; STD: 1.77 vs. 1.14 brpm and RMSD: 1.83 vs. 1.16 brpm). This may be a result of the more challenging nature of the GCF patients compared to the healthy subjects studied in this earlier work. In order to assess the effects of intra-patient dependency within the analysis, we investigated the inter-patient variability of the results. The individual statistics for each subject were computed and it was found that the mean RMSD was 1.73 brpm and standard deviation of the RMSD’s was 1.28 brpm. This supports the likelihood of an acceptably small intra-patient variability in the difference between RR_oxi_ and the reference.

Importantly, this performance was accomplished using a single sensor that combines the ability to monitor RR, arterial oxygen saturation (SpO_2_), and pulse rate; these findings highlight a unique, clinically useful approach to monitoring multiple respiratory variables in a continuous, non-invasive, and easy-to-use manner. An advantage of the use of a single senor for SpO_2_, HR and RR is that it potentially reduces the number of alarm modes. For example, a single “sensor off” or “motion artifact” alarm could cover all three parameters, whereas in two separate devices there is a likelihood of significant increased false alarm rate, with a corresponding likelihood of increased alarm fatigue and workflow disruption [46].

The significance of the magnitude of absolute error associated with respiratory rate depends on the on the true rate. The results published here mostly lie in a central range of respiration between approximately 10 and 20 brpm. We performed sub-analyses of the results for RR <10 brpm and >20 brpm and found the mean, SD and RMSD to be 1.07, 2.42, 2.65 brpm respectively for rates <10 brpm and 1.33, 1.76, 2.20 brpm respectively for rates greater than 20 brpm. These sub-analyses involved 6.1 and 17.1 % data points respectively of the total collected. These values are slightly greater than for the whole data set. It is important, however, not to over-interpret these results as there are relatively few data at these extremes. Our group is very aware of the importance of the algorithm performance at the extremes and have ongoing work considering patient groups that cover these regions [55, 56].

The physiological processes that both enable and confound the measurement of respiratory rate appear well understood in this space and link to the wider literature on the causes of erroneous PPG components including: vasotone, vasomotion, posture, patient motion, temperature, metabolic state, pain, drug administration, lung compliance, upper airway obstruction, edema, heart rate, respiratory rate, catheterization, ablation, the venous blood component and arrhythmia. These are documented more fully in the work of others [2, 16, 37–45]. There are little data on GCF monitoring of the PPG for RR as this is an area of care where continuous oximeter monitoring is not regularly carried out. However, we have observed in our own work that operating room data exhibits considerable PPG artifact from: motion, drug administration, vasomotion, administration of fluids, heart rate changes, etc., whereas the GCF is a more benign environment. If continuous PPG-based RR were to gain traction in the GCF, we expect that much of the signal would be of good quality with intermittent instances of severe artifact due to patient motion: voluntary or assisted.

It is worth noting that our wavelet-based algorithm does not tend to exhibit the erroneous low rates posted across the range of reference rates prevalent in the work Karlen et al. [18]. We believe that this is due to increased flexibility in identifying and partitioning respiratory components inherent in our approach over their smart-fusion, frequency spectrum based approach. Our results compare favourably with those reported by other groups [13–18, 21–29, 31–36]. However, such comparisons should be considered carefully as the results are highly dependent on the characteristics of the raw signal and its manipulation, exclusion criteria, manual selection of data (if applicable), the patient group studied and, of course, the algorithmic implementation (including pre-processing, processing and post-processing steps). The determination of a clinically useful physiological parameter is therefore a distinctly non-trivial task. An important aspect of our work is that it targets the development of a fully-automated algorithm capable of coping with the extremes of data characteristics in the clinical environment: i.e. the RR_oxi_ values generated are those that would be displayed on the device screen to the clinician. A sophisticated algorithmic infrastructure is therefore required to take the raw biosignal from the hardware, process it, present it to the core algorithm, then apply further post-processing to the output in order to produce a value with the integrity necessary for display on the screen of a medical monitoring device [2].

It is well-established that many patient deaths on the hospital GCF may be prevented, at least in part, through more vigilant monitoring aimed at detecting clinically meaningful antecedents to patient deterioration [8]. For example, Hodgetts et al. [9] reported in a root-cause analysis that approximately 80 % of the cardiac arrests occurring on the GCF were preventable. It has been reported that approximately 40 % of such alterations are considered respiratory in nature, underscoring the importance of attentive respiratory monitoring in this setting [20]. Despite this, it has been suggested that upon the arrival of a hospital rapid response team, up-to-date vital signs, such as RR, are not available for three out of four patients [4]. Clearly, providing this information in a continuous and timely manner to clinicians could provide the foundation for improved patient outcomes on the GCF. A critical factor contributing to respiratory distress on the GCF is the administration of opioid analgesia and associated respiratory depression [3]. Consequently, the Anesthesia Patient Safety Foundation has issued guidance suggesting that for patients receiving post-operative opioid analgesia administration, vital sign monitoring should occur with increased frequency [19]. Thus, monitoring RR continuously may offer an avenue to specifically reduce the deleterious impact of opioid-induced respiratory depression.

Despite the overwhelming importance of a patient’s respiratory status while on the GCF, manual observation remains the standard of care for assessment of RR. It is clear to see that this intermittent approach is lacking because it leaves substantial periods of time in which the patient’s respiratory status is unmonitored. Given the rapidity with which a patient’s respiratory status may devolve, critical clinical information during these unmonitored periods of time leave the patient susceptible to the untoward clinical complications mentioned above. In addition to patient safety considerations, there is also a clinical burden placed on the staff to monitor RR at periodic intervals. By establishing a means through which RR can be monitored continuously, in conjunction with pulse oximetry from a single sensor site, our algorithm provides a mechanism to potentially improve patient outcomes on the GCF while improving compliance with vital sign monitoring requirements.

### Concluding remarks

Our results demonstrate that the RR_oxi_ algorithm is a potentially viable technological approach for monitoring RR in a diverse GCF patient population. Currently, pulse oximeters use the differential absorption of red and infrared light between oxygenated hemoglobin and deoxygenated hemoglobin to provide a measure of oxygen saturation, with heart rate also provided. These devices do not measure RR, and will only detect inadequate respiration after hypoxia has occurred. Hence, pulse oximetry may be considered a lagging indicator of evolving respiratory complications, limiting its efficacy in this domain. However, the combination of pulse oximetry with RR, in a single sensor, may provide earlier indication of evolving respiratory compromise. We believe that the RR_oxi_ algorithm would provide this vital information by offering the capability to monitor RR via a probe that is routinely attached to patients in many clinical situations, thus enhancing patient safety and facilitating reduced clinical workflow with combined RR and oxygen saturation monitoring.

## References

[1] Addison PS (2002). The illustrated wavelet transform handbook.

[2] Addison PS, Watson JN, Mestek ML, Mecca RS (2012). Developing an algorithm for pulse oximetry derived respiratory rate (RRoxi): a healthy volunteer study. J Clin Monit Comput..

[3] Bowker L, Stewart K (1999). Predicting unsuccessful cardiopulmonary resuscitation (CPR): a comparison of three morbidity scores. Resuscitation.

[4] Chen J, Hillman K, Bellomo R, Flabouris A, Finfer S, Cretikos M (2009). The impact of introducing medical emergency team system on the documentations of vital signs. Resuscitation.

[5] Dahan A, Aarts L, Smith TW (2010). Incidence, reversal, and prevention of opioid-induced respiratory depression. Anesthesiology.

[6] Folke M, Cernerud L, Ekstrom M, Hok B (2003). Critical review of non-invasive respiratory monitoring in medical care. Med Biol Eng Comput..

[7] Green SM, Pershad J (2010). Should capnographic monitoring be standard practice during emergency department procedural sedation and analgesia? Pro and con. Ann Emerg Med..

[8] Hillman KM, Bristow PJ, Chey T (2001). Antecedents to hospital deaths. Intern Med J..

[9] Hodgetts TJ, Kenward G, Vlackonikolis I (2002). Incidence, location and reasons for avoidable in-hospital cardiac arrest in a district general hospital. Resuscitation.

[10] Leonard P, Beattie TF, Addison PS, Watson JN (2003). Standard pulse oximeters can be used to monitor respiratory rate. Emerg Med J..

[11] Leonard PA, Clifton D, Addison PS, Watson JN, Beattie T (2006). An automated algorithm for determining respiratory rate by photoplethysmogram in children. Acta Paediatr..

[12] Leonard PA, Douglas JG, Grubb NR, Clifton D, Addison PS, Watson JN (2006). A fully automated algorithm for the determination of respiratory rate from the photoplethysmogram. J Clin Monit Comput..

[13] Dash S, Shelley KH, Silverman DG, Chon KH (2010). Estimation of respiratory rate from ECG, photoplethysmogram, and piezoelectric pulse transducer signals: a comparative study of time-frequency methods. IEEE Trans Biomed Eng..

[14] Leuvan CH, Mitchell I (2008). Missed opportunities? An observational study of vital sign measurements. Crit Care Resusc..

[15] Lindberg LG, Ugnell H, Oberg PA (1992). Monitoring of respiratory and heart rates using a fibre-optic sensor. Med Biol Eng Comput..

[16] Nilsson L, Johansson A, Kalman S (2003). Macrocirculation is not the sole determinant of respiratory induced variations in the reflection mode photoplethysmographic signal. Physiol Meas..

[17] Lazaro J, Gil E, Bailon R, Minchole A, Laguna P (2013). Deriving respiration from photoplethysmographic pulse width. Med Biol Eng Comput..

[18] Karlen W, Raman S, Ansermino JM, Dumont GA (2013). Multiparameter respiratory rate estimation from the photoplethysmogram. IEEE Trans Biomed Eng..

[19] Pasero C, McCaffery M (2011). Pain assessment and pharmacologic management.

[20] Schein RM, Hazday N, Pena M, Ruben BH, Sprung CL (1990). Clinical antecedents to in-hospital cardiopulmonary arrest. Chest.

[21] Chon KH, Dash S, Ju K (2009). Estimation of respiratory rate from photoplethysmogram data using time-frequency spectral estimation. IEEE Trans Biomed Eng..

[22] Clifton D, Douglas JG, Addison PS, Watson JN (2007). Measurement of respiratory rate from the photoplethysmogram in chest clinic patients. J Clin Monit Comput..

[23] Fleming S, Tarassenko L, Thompson M, Mant D. Non-invasive measurement of respiratory rate in children using the photoplethysmogram. Conf Proc IEEE Eng Med Biol Soc. 2008; 1886–1889. 10.1109/IEMBS.2008.4649554 19163057

[24] Fleming SG, Tarassenko L (2007). A comparison of signal processing techniques for the extraction of breathing rate from the photoplethysmogram. Intern J Biol Med Sci..

[25] Foo JY, Wilson SJ (2005). Estimation of breathing interval from the photoplethysmographic signals in children. Physiol Meas..

[26] Johansson A (2003). Neural network for photoplethysmographic respiratory rate monitoring. Med Biol Eng Comput..

[27] Johansson A, Oberg PA, Sedin G (1999). Monitoring of heart and respiratory rates in newborn infants using a new photoplethysmographic technique. J Clin Monit Comput..

[28] Johnston WS, Mendelson Y (2004). Extracting breathing rate information from a wearable reflectance pulse oximeter sensor. Conf Proc IEEE Eng Med Biol Soc..

[29] Lee J, Chon KH (2010). Respiratory rate extraction via an autoregressive model using the optimal parameter search criterion. Ann Biomed Eng..

[30] Leonard P, Grubb NR, Addison PS, Clifton D, Watson JN (2004). An algorithm for the detection of individual breaths from the pulse oximeter waveform. J Clin Monit Comput..

[31] Nilsson L, Johansson A, Kalman S (2000). Monitoring of respiratory rate in postoperative care using a new photoplethysmographic technique. J Clin Monit Comput..

[32] Nilsson L, Johansson A, Kalman S (2005). Respiration can be monitored by photoplethysmography with high sensitivity and specificity regardless of anaesthesia and ventilatory mode. Acta Anaesthesiol Scand..

[33] Olsson E, Ugnell H, Oberg PA, Sedin G (2000). Photoplethysmography for simultaneous recording of heart and respiratory rates in newborn infants. Acta Paediatr..

[34] Shelley KH, Awad AA, Stout RG, Silverman DG (2006). The use of joint time frequency analysis to quantify the effect of ventilation on the pulse oximeter waveform. J Clin Monit Comput..

[35] Wertheim D, Olden C, Savage E, Seddon P (2009). Extracting respiratory data from pulse oximeter plethysmogram traces in newborn infants. Arch Dis Child Fetal Neonatal Ed..

[36] Zhou Y, Zheng Y, Wang C, Yuan J (2006). Extraction of respiratory activity from photoplethysmographic signals based on an independent component analysis technique: Preliminary report. Instrum Sci Technol..

[37] Alian AA, Shelley KH. Respiratory Physiology and the impact of different modes of ventilation on the photoplethysmographic waveform. 2012;2236–2254. 10.3390/s120202236 PMC3304164 22438762

[38] Allen J, Frame JR, Murray A (2002). Microvascular blood flow and skin temperature changes in the fingers following a deep inspiratory gasp. Physiol Meas..

[39] Delerme S, Renault R, Le Mannach Y, Lvovschi V, Bendahou M, Riou B, Ray P (2007). Variations of pulse oximetry plethysmographic waveform amplitude induced by passive leg raising in spontaneously breathing volunteers. Am J Emerg Med..

[40] Hamunen K, Kontinen V, Hakala E, Talke P, Paloheimo M, Kalso E (2012). Effect of pain on autonomic nervous system indices derived from photoplethysmography in healthy volunteers. Br J Anaesth..

[41] Knorr-Chung BR, McGrath SP, Blike GT (2008). Identifying airway obstructions using photoplethysmography (PPG). J Clin Monit Comput..

[42] Reisner A, Shaltis PA, McCombie D, Asada HH (2008). Utility of the photoplethysmogram in circulatory monitoring. Anesthesiology.

[43] Selvaraj N, Jaryal AK, Santhosh J, Deepak KK, Anand S (2009). Influence of respiratory rate on the variability of blood volume pulse characteristics. J Med Eng Technol..

[44] Shah A, Shelley KH (2013). Is pulse oximetry an essential tool or just another distraction? The role of the pulse oximeter in modern anesthesia care. J Clin Monit Comput..

[45] Shelley KH (2007). Photoplethysmography: beyond the calculation of arterial oxygen saturation and heart rate. Anesth Analg..

[46] ECRI Institute. Top 10 Health Technology Hazards For 2014. Adapted from: Health Devices. ECRI Institute, Plymouth Meeting, PA. 2013;42(11). 24358513

[47] Meredith DJ, Clifton D, Charlton P, Brooks J, Pugh CW, Tarassenko L (2013). Photoplethysmographic determination of respiratory rate: a review of current literature. J Med Eng Technol..

[48] Addison PS, Watson JN. Wavelet-based analysis of pulse oximeter signals. US Patent 7,035,679. 2006.

[49] Addison PS, Watson JN, Clifton D. Methods and systems for discriminating bands in scalograms. US Patent 8,077,297. 2011.

[50] Watson JN, Addison PS. Methods and systems for filtering a signal according to a signal model and continuous wavelet transform techniques. US Patent 8,235,911. 2012.

[51] Watson JN, Addison PS, Clifton D. Systems and methods for ridge selection in scalograms. US Patent 8,295,567. 2012.

[52] Watson JN, Addison PS, Van Slyke BM. Systems and methods for estimating values of a continuous wavelet transform. US Patent 8,346,333. 2013.

[53] McGonigle S, Addison PS, Ochs J, Watson JN. Systems and methods for determining respiration information from a photoplethysmograph. US Patent Application 20130079606. 2013.

[54] ISO 80601-2-61:2011. Medical electrical equipment—Part 2-61: particular requirements for basic safety and essential performance of pulse oximeter equipment. International Organization for Standardization, 2011.

[55] Mestek ML, Ochs JP, Addison PS, Neitenbach AM, Bergese SD, Kelley SD (2013). Accuracy of continuous non-invasive respiratory rate derived from pulse oximetry in patients with high respiratory rates. Abstracts of papers presented at the, 2013 annual meeting of the society for technology in anesthesia (STA). Anesth Analg Suppl..

[56] Kelley SD, Neitenbach AM, Kinney AR, Mestek ML. Detection of opioid-induced respiratory depression with pulse oximetry derived respiratory rate monitoring. Abstract A095. Am Soc Anesth. 2012;Washington, DC.

[57] Mestek ML, Addison PS, Watson JN, Neitenbach AM, Ochs JP. Accuracy of continuous non-invasive respiratory rate derived from pulse oximetry during coached breathing. I.A.M.P.O.V. 2012 Symposium, Yale University, New Haven, CT, USA. 29 June–1 July 2012.

[58] Addison PS, Mestek ML, Watson JN, Neitenbach AM, Ochs JP. Continuous respiration rate derived from pulse oximetry during cold-room hypoxia. I.A.M.P.O.V. 2012 Symposium, Yale University, New Haven, CT, USA. 29 June–1 July 2012.

[59] Mestek ML, Addison PS, Neitenbach AM, Bergese SD, Kelley SD. Accuracy of continuous non-invasive respiratory rate derived from pulse oximetry in the postanesthesia care unit. Abstract 094, American Soc Anesth, Annual Meeting, October 13–17, 2012; Washington, DC.

[60] Mestek ML, Addison PS, Kinney AR, Kelley SD. Accuracy of continuous non-invasive respiratory rate derived from pulse oximetry in obese subjects. Abstract A561, American Soc. Anesth., Annual Meeting, October 13–17, 2012; Washington, DC.

[61] Mestek ML, Addison PS, Neitenbach AM, Bergese SD, Kelley SD. Accuracy of continuous noninvasive respiratory rate derived from pulse oximetry in congestive heart failure patients. Chest 2012;142 (4_MeetingAbstracts):113A.

[62] Mestek ML, Addison PS, Neitenbach AM, Bergese SD, Kelley SD. Accuracy of continuous noninvasive respiratory rate derived from pulse oximetry in chronic obstructive pulmonary disease patients. Chest 2012;142 (4_MeetingAbstracts): 671A.

